# Aspects of Dental Occlusion in Young University Students with Temporomandibular Dysfunction

**DOI:** 10.3390/diagnostics16213608

**Published:** 2026-11-01

**Authors:** Ștefana Meșină-Dică, Veronica Mercuț, Virginia Maria Rădulescu, Diana-Elena Vlăduțu, Cătălina Măgureanu-Murariu, Mihaela-Roxana Boțilă, Monica Scrieciu, Mihnea Meșină, Alexandra Maria Rădoi, Petre-Costin Mărășescu

**Affiliations:** 1 Department of Prosthetic Dentistry, University of Medicine and Pharmacy of Craiova, 200349 Craiova, Romania; stefana.dica@umfcv.ro (Ș.M.-D.); diana.vladutu@umfcv.ro (D.-E.V.); monica.scrieciu@umfcv.ro (M.S.); alexandra.radoi@umfcv.ro (A.M.R.); marasescup@yahoo.com (P.-C.M.); 2 Dental Medicine Research Centre (CCMD), University of Medicine and Pharmacy of Craiova, 200349 Craiova, Romania; 3 Department of Medical Informatics and Biostatistics, Faculty of Medicine, University of Medicine and Pharmacy of Craiova, 200349 Craiova, Romania; 4 Department of Prosthodontics, Faculty of Dentistry, “Carol Davila” University of Medicine and Pharmacy, 020021 Bucharest, Romania; catalina.magureanu.murariu@umfcd.ro; 5 Department of Urology, University of Medicine and Pharmacy of Craiova, 200349 Craiova, Romania; mihneamesina@yahoo.com

**Keywords:** T-Scan system, temporomandibular dysfunction, Fonseca Anamnestic Index

## Abstract

**Background/Objectives**: Temporomandibular dysfunction (TMD) has a multifactorial aetiology, and the contribution of dental occlusion remains controversial. The objectives of the study were to estimate the proportion of students at the Faculty of Dentistry in Craiova with Fonseca Anamnestic Index (FAI) scores suggestive of TMD-related manifestations and to highlight possible associations between dental occlusion and FAI scores in a selected subset. **Methods**: A total of 106 students completed the Romanian version of the FAI. Three participants declined further participation after completing the questionnaire and before FAI scoring; their responses were not scored. The remaining 103 questionnaires were included in the FAI analysis. Following the screening, a study group of 20 students was selected from the upper end of the recorded score distribution, and a control group of 20 was selected from the lower end; 17 controls scored zero and three scored five. Static and dynamic occlusal parameters were assessed with T-Scan III where valid recordings were available. Between-group comparisons and Spearman correlations were performed, with Benjamini–Hochberg false discovery rate adjustment within defined exploratory test families. **Results**: The study showed that 43 of 103 students (41.75%) had FAI scores suggestive of TMD-related manifestations after the discrepant totals had been reconciled with the source questionnaires. In the selected groups, maximum intercuspation occlusion time (*p* < 0.001; q = 0.015), protrusive disclusion time (*p* = 0.003; q = 0.020), and active premature contacts during right and left lateral excursions (both *p* = 0.002; q = 0.015) differed after adjustment. **Conclusions**: Occlusal assessment with the T-Scan III System showed that differences between the selected study and control groups were more evident for dynamic parameters than for static occlusal-force distribution. The FAI does not establish a clinical TMD diagnosis, and the exploratory cross-sectional design does not support causal or therapeutic conclusions.

## 1. Introduction

Temporomandibular dysfunction (TMD) comprises a range of musculoskeletal conditions affecting the masticatory muscles, temporomandibular joints (TMJs), and surrounding structures [1,2,3]. More than 30 conditions have been identified within the TMD spectrum [2]. Common DC/TMD diagnoses include arthralgia, myalgia, local myalgia, myofascial pain with or without referral, articular disc disorders, degenerative joint disease, subluxation, and headache [4]. In addition to these clinical manifestations, TMD has a negative impact on quality of life [5,6].

The most frequently reported TMD manifestations are myalgia (37.2%), joint sounds/clicking (29.8%), and arthralgia (16.8%), whereas limited mouth opening/locking is reported least frequently (8.1%) [7]. TMD is among the most common diagnoses associated with orofacial pain in dental practice. Horst et al. (2015) reported that one in six patients who had visited a general dentist during the preceding year experienced orofacial pain [8]. Pain in the masticatory muscles and TMJ region was reported as frequently as dental pain or pain arising from the surrounding oral tissues [8].

A meta-analysis estimated that almost one-third of the global population (29.5%) has TMD, with women being more frequently affected than men [7]. Prevalence among individuals younger than 18 years is 38.5%, compared with 34.1% among those aged 18 years or older [7]. TMD is more prevalent in Europe (33.8%), Asia (27.9%), and South America (27.3%), and least prevalent in North America (19.4%). Lekaviciute et al. (2024) estimated that 50% of adults experience TMD-related symptoms [9].

The aetiology of TMD is multifactorial and involves biomechanical, neuromuscular, biopsychosocial, and biological factors [10]. Occlusal disharmonies have been considered an aetiological factor, although the extent and mechanisms through which they may contribute to TMD remain unclear [11,12]. Okeson described a complex interaction among occlusal status, trauma, emotional stress, deep pain input, and parafunctional activities [13]. Moreno-Hay and Okeson found no indication that permanent alteration of the occlusal vertical dimension produces long-lasting TMD symptoms [14]. Occlusal instability may overload the masticatory muscles and affect the TMJ [15]. Cao et al. (2009) found that experimental occlusal interference induced long-term mechanical hyperalgesia in rat masticatory muscles, with central sensitization implicated in its maintenance [16]. Nevertheless, the relationship between occlusion and TMD remains controversial [17,18], and some occlusal characteristics may be consequences rather than causes of TMD [19,20]. Türp et al. (2008) proposed that painful TMD may disturb mandibular dynamics and static positions, thereby inducing occlusal dysfunction [21]. Kalladka et al. (2022) found no robust primary research establishing a cause-and-effect relationship between dental occlusion and TMD, while noting that several TMD conditions may lead to secondary changes in dental occlusion [22]. The potential role of orthodontic treatment as a risk factor for TMD also remains debated [17].

Research on digitally guided occlusal adjustment using the T-Scan system has reported an improvement in TMD-associated muscular symptoms following a reduction in disclusion time [23,24,25]. In a multicenter randomized study, Thumati et al. observed improvements in pain and functional limitations—accompanied by electromyographic changes—compared to the control intervention [23]. The reduction in masseter and temporalis activity during mandibular excursions following the shortening of disclusion time supports the involvement of a neuromuscular response [26]; other studies have reported a reduction in myofascial pain [24,25].

This study estimated the proportion of assessed dental students with FAI scores suggestive of TMD-related manifestations and explored associations between FAI scores and T-Scan III occlusal measures in participants selected for instrumental assessment.

Against this background, the present study aimed to estimate the proportion of assessed dental students at the Faculty of Dentistry in Craiova with FAI scores suggestive of TMD-related manifestations, to explore associations between FAI scores and T-Scan measures in the selected subset, and to describe self-reported previous orthodontic and occlusal dental treatment.

## 2. Materials and Methods

### 2.1. Study Design

The study was conducted between 3 February 2026 and 14 September 2026 at the Prosthodontics Clinic of the Faculty of Dentistry in Craiova. A total of 106 fourth-year students completed the questionnaire; three declined further participation after questionnaire completion and before FAI scoring, leaving 103 questionnaires for analysis. TMD-related manifestations were screened using the Romanian version of the Fonseca Anamnestic Index (FAI) [27], while dental occlusion was evaluated clinically and with the T-Scan III system (Tekscan Inc., South Boston, MA, USA) [28].

The study was approved by the Ethics and Deontology Committee of the University of Medicine and Pharmacy of Craiova (No. 11/03.02.2026) and was conducted in accordance with the Declaration of Helsinki [29].

### 2.2. Formation of the Study Groups

Fourth-year students from the Faculty of Dentistry, who had an intermediate level of knowledge of occlusion and TMD, were considered for inclusion. The following inclusion and exclusion criteria were applied:

Inclusion criteria: students of either sex who agreed to participate and were interested in the assessment of dental occlusion and the presence of TMD signs.

Exclusion criteria: students who declined participation, students absent on recording days, students wearing orthodontic appliances, and students without stable occlusion, operationally defined as absence of occlusal stops in at least one of the four posterior supporting zones (i.e., not meeting Eichner class A criteria) [30].

Of the 106 students who completed the questionnaire, three declined further participation before FAI scoring; their responses were not scored or included in the FAI analysis. The remaining 103 questionnaires were scored, and 40 students were selected for instrumental assessment based on their FAI scores.

#### 2.2.1. Identification of TMD

The Romanian version of the Fonseca questionnaire was administered during a teaching session [27]. Two additional questions recorded participants’ previous treatment history. The first asked whether they had previously undergone orthodontic treatment. The second asked (translated from Romanian): “Have you had dental treatment that involved the occlusal surface of your teeth?” This item captured self-reported treatment history; the questionnaire did not specify the type of treatment. For participants who reported such treatment and any symptoms, a follow-up question asked: “If you reported any symptoms, did they begin before or after the dental treatment involving the occlusal surface?”. Participants could also indicate that they could not specify the temporal relationship.

The Fonseca Anamnestic Index is a multidimensional instrument widely used for epidemiological screening of TMD [31,32]. It comprises 10 closed-ended questions assessing pain in the TMJ, masticatory muscles, neck, and head, functional limitations, joint sounds, parafunctional habits, and emotional stress [33].

Each response is scored as “No” (0 points), “Sometimes” (5 points), or “Yes” (10 points). The sum ranges from 0 to 100 and indicates no FAI manifestations (0–15), mild (20–40), moderate (45–65), or severe (70–100) manifestations. These screening categories do not establish a clinical TMD diagnosis.

Participants were selected from the upper and lower ends of the recorded FAI-score distribution for instrumental assessment: the analysed study group consisted of IDs 1–20 and the analysed control group of IDs 84–103. Seventeen analysed controls scored zero and three scored five. Tie-breaking and the exclusion of another control-coded participant (ID 83) require confirmation from the clinical team.

#### 2.2.2. Assessment of Dental Occlusion Using the T-Scan System

All 40 participants included in the study and control groups underwent clinical examination, with particular attention to the inclusion and exclusion criteria. Computerised T-Scan analysis was then performed to assess static and dynamic occlusal parameters. The system records and evaluates contacts between the maxillary and mandibular teeth, including occlusal-force distribution, the dynamics of occlusal relationships, and their duration. The T-Scan III system (Tekscan Inc., South Boston, MA, USA) was used.

The T-Scan III system consists of a sensor, sensor support, handpiece, and software. The U-shaped sensor is inserted into the handheld device, connected to a computer via USB, and positioned intraorally between the occlusal surfaces. T-Scan III enables assessment of occlusal-force distribution for individual teeth, each hemiarch, or selected arch segments, as well as changes in occlusal forces during mandibular movements [28,34]. Recordings are stored digitally and can be replayed as time-based videos [28]. The sensor is a 60-µm polyester film [35] available in “Small” and “Large” sizes [36]. Attached to the handpiece, it samples at millisecond intervals. The system analyses both timing and force [37,38], recording the timing of occlusal contacts and the relative force generated by each contact.

For recording, participants were seated in a physiological position, with the head aligned with the spine and the Frankfurt plane horizontal.

Dynamic occlusal recordings were obtained using the T-Scan system, starting from maximum intercuspation. Participants were asked to perform protrusion until the incisors reached an edge-to-edge position, as well as right and left laterotrusion until an edge-to-edge position was reached by the canines or by the group of teeth providing guidance, as applicable. Occlusal contacts were evaluated throughout each movement and at its end [39]. For each movement, participants first maintained maximum intercuspation for 3–5 s and then performed the movement, following the T-Scan recording protocol described by Chutchalermpan et al. [40].

The software was started and the handpiece connected to the computer. An appropriately sized sensor was selected and positioned between the maxillary central incisors. For calibration, an initial recording was obtained in maximum intercuspation (MI). Participants were instructed to occlude with maximum intensity for 3 s, allowing adjustment of the response threshold using the “Sensitivity Adjustment Selection” function. Recording was initiated by pressing the button on the handpiece, after which the software automatically generated a graphical representation of the dental arch.

The following occlusal parameters were assessed: in MI, distribution of occlusal forces across the right and left hemiarches, occlusion time, and disclusion time; during protrusion, presence of incisal guidance, active and inactive interferences, active and inactive premature contacts, and disclusion time; and during right and left lateral excursions, type of guidance, active and inactive interferences, active and inactive premature contacts in the lateral edge-to-edge position, and disclusion time.

A premature contact was defined as a contact between opposing teeth that was present at the end of the mandibular movement and associated with mandibular deviation. To identify premature contacts, the opposing tooth pairs making the first contact at the end of the mandibular movement were recorded [41]. Occlusal interferences were defined as contacts between opposing teeth that occurred during mandibular movements and were associated with deviation of the mandibular trajectory. Protrusive, laterotrusive, and mediotrusive interferences were evaluated. Based on the T-Scan recordings, opposing tooth pairs making occlusal contact during the movement were considered interferences, except for the teeth providing guidance [28].

#### 2.2.3. Statistical Analysis

The ten FAI items were scored 0, 5, or 10 and summed to obtain a total ranging from 0 to 100. Item sums were checked against the recorded totals before analysis. FAI categories were defined as no manifestations (0–15), mild (20–40), moderate (45–65), and severe (70–100). The proportion above the screening threshold was calculated in the 103 students and reported with a Wilson 95% confidence interval. The final instrumental sample comprised the 20 examined students with higher recorded FAI scores (IDs 1–20) and 20 examined students with lower scores (IDs 84–103); these were the actually examined groups, irrespective of corrections to recorded totals. The group difference in FAI score and comparisons between its constituent items were treated as descriptive features of selection. Continuous variables are summarised as mean ± standard deviation and, where informative, median and interquartile range. Between-group comparisons used two-sided Mann–Whitney U tests, except for the added comparison of mean individual force deviation from 50:50, which used a two-sided Welch t-test; binary variables used two-sided Fisher exact tests. At maximum intercuspation, individual absolute deviation from a 50:50 bilateral force distribution was calculated in percentage points as 50 × |R − L|/(R + L), using the recorded right (R) and left (L) force values. Both values were required; the between-group mean difference was reported with a Welch 95% confidence interval. Categorical guidance types were described without formal group testing. The sex-by-FAI-category table was evaluated using a fixed-margin Monte Carlo test based on 100,000 random tables because expected cell counts were small. Associations between summed FAI score and occlusal measures in the selected sample were estimated using Spearman ρ. Effect sizes for the other continuous-group comparisons are rank-biserial correlations with stratified percentile bootstrap 95% confidence intervals (1600 replicates); binary comparisons show risk differences with Newcombe–Wilson 95% confidence intervals. Correlation intervals were estimated with 1600 bootstrap replicates stratified by selected group. Bootstrap intervals describe conditional precision in this selected sample and do not remove selection bias. Analyses used available valid observations separately for each variable. A missing recording was distinguished from a movement that could not be performed, for which a movement duration was undefined rather than zero. The left-force entry for ID 95 was excluded from the original side-specific comparison because its recorded scale was initially unclear; its subsequently verified percentage value was included in the added bilateral deviation analysis. Exploratory multiplicity was addressed using the Benjamini–Hochberg procedure within four declared families: 23 study–control comparisons, 21 FAI correlations, 19 Q7 comparisons, and 19 Q9 comparisons. Both nominal *p*-values and adjusted q-values are reported. Analyses of Q7 and Q9 were exploratory because these items contribute to the FAI score used to select the groups. The conventional tests can be run using the supplied IBM SPSS Statistics 26 syntax; the reported numerical results, confidence intervals and fixed-margin Monte Carlo check were calculated with the accompanying verification script. Statistical findings were interpreted in conjunction with effect magnitude and precision, without causal inference. IBM SPSS Statistics version 26.0 was used for standard tabulations and tests; the fixed-margin Monte Carlo calculation, bootstrap intervals, and exact confidence intervals were reproduced with Python 3.12. The source questionnaires confirmed the item responses for IDs 10, 19, 29, 32 and 65. Their recorded totals were corrected to match the ten-item sums (25, 40, 30, 10 and 5 points, respectively) before the analyses were finalised.

## 3. Results

### 3.1. FAI Screening Findings in the Initial Sample

There were 103 students with unique identifiers (61 women and 42 men; mean age 24.15 ± 3.40 years). After checking five source questionnaires, the recorded totals for IDs 10, 19, 29, 32 and 65 were corrected to match the confirmed item responses. Based on the item sums, the mean FAI score was 17.14 ± 15.79 (median 15; interquartile range 5–25). Forty-three students (41.75%; Wilson 95% CI 32.69–51.40%) scored above the FAI threshold for TMD-related manifestations: 36 mild, six moderate, and one severe (Table 1, Table 2 and Table 3). This is a screening proportion in the observed student sample, not a clinically confirmed population prevalence. The sex-by-category association was not supported by the fixed-margin Monte Carlo analysis (*p* ≈ 0.530). Three of the 106 students who completed the questionnaire declined further participation before FAI scoring; their responses were not included in the FAI analysis.

#### 3.1.1. Distribution of FAI Screening Categories

Using the sums of the ten item responses, 60/103 participants scored 0–15, 36 scored 20–40, six scored 45–65, and one scored 70–100. The sex-by-category table did not show evidence of an association in the fixed-margin Monte Carlo analysis (*p* ≈ 0.530; Table 2).

After source-form verification, 43/103 students (41.75%; Wilson 95% CI 32.69–51.40%) had an FAI score suggestive of TMD-related manifestations. The corrected total-score field agreed with the item sums for all 103 students.

#### 3.1.2. Distribution of FAI Item Responses

Responses to the 10 Fonseca items showed considerable variability in TMD-related symptoms.

**Figure 1:**
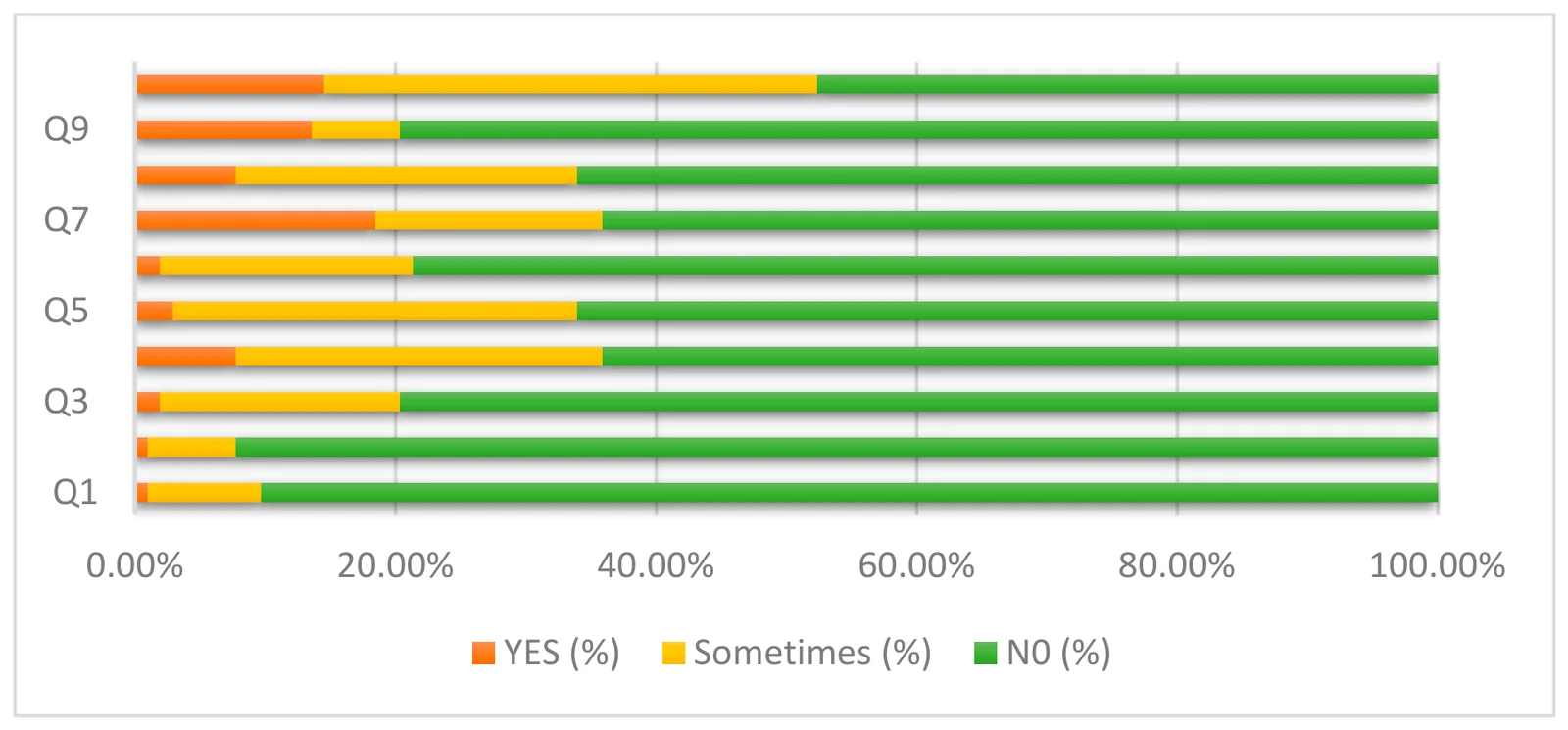
Distribution of Fonseca Anamnestic Index responses in the initial sample (*n* = 103).

The most frequently reported manifestation was feeling tense or anxious (Q10: 54/103, 52.43% positive), whereas difficulty moving the mandible from side to side (Q2: 8/103, 7.77%) and difficulty opening widely (Q1: 10/103, 9.71%) were least frequent (Figure 1). Individual item responses are descriptive and do not constitute a diagnosis.

Frequent headaches (Q4) and TMJ sounds during chewing or wide mouth opening (Q7) were the next most common findings, each with a positive-response rate of 35.92% (*n* = 37). Q7 also had the highest proportion of “Yes” responses across the questionnaire (18.45%).

Neck pain or stiffness (Q5, 33.98%, *n* = 35) and clenching or grinding of the teeth (Q8, 33.98%, *n* = 35) followed, with “Sometimes” responses predominating over “Yes” responses (31.07% vs. 2.91% and 26.21% vs. 7.77%, respectively).

Symptoms of moderate frequency included pain or fatigue in the masticatory muscles during chewing (Q3, 20.39%, *n* = 21) and pain in the ear region or TMJ discomfort (Q6, 21.36%, *n* = 22), followed by the perception that the teeth did not contact correctly (Q9, 20.39%, *n* = 21), for which 13.59% answered “Yes”.

Statistical interpretation: the most frequent positive responses were recorded for Q10 (54/103, 52.43%), followed by Q4 and Q7 (37/103, 35.92% each). This pattern indicates prominent tension/anxiety, headache, and joint sounds in the symptom profile.

### 3.2. Characteristics and FAI Findings in the Final Study and Control Groups

#### 3.2.1. Formation and Characteristics of the Final Groups

Twenty students from each end of the recorded FAI-score distribution underwent selection for instrumental assessment. After recalculating item sums, the study-group mean was 41.75 ± 13.40 (median 37.50), whereas the control-group mean was 0.75 ± 1.83 (median 0); 17 controls scored zero and three scored five. ID 83 was coded as a control and had three populated left-lateral T-Scan fields, but was not included among the 20 analysed controls; the reason requires clinical confirmation. The group selection was performed on recorded totals after reconciling the recorded totals with the confirmed item responses. One examined study-group participant scored 25, while six unexamined participants scored 30. Group contrasts in FAI score or constituent items therefore document the sampling strategy rather than provide independent evidence of an occlusal association (Table 4, Table 5 and Table 6). Previous treatment of occlusal surfaces was reported by 12/20 study participants and 5/20 controls (*p* = 0.054; q = 0.088 in the group-comparison family; Table 7).

The sex distribution of these deliberately selected groups differed numerically, but the effective sample is small, and the contrast does not describe the whole cohort.

#### 3.2.2. FAI Screening Categories in the Final Groups

The study group had higher FAI screening scores than the controls by design. The control group included 17 participants with a score of zero and three with a score of five. A questionnaire category does not constitute a confirmed clinical diagnosis.

The FAI score difference reflects selection on that same score and is not independent evidence of a biological association.

#### 3.2.3. Comparison of FAI Item Scores Between the Study and Control Groups

**Figure 2:**
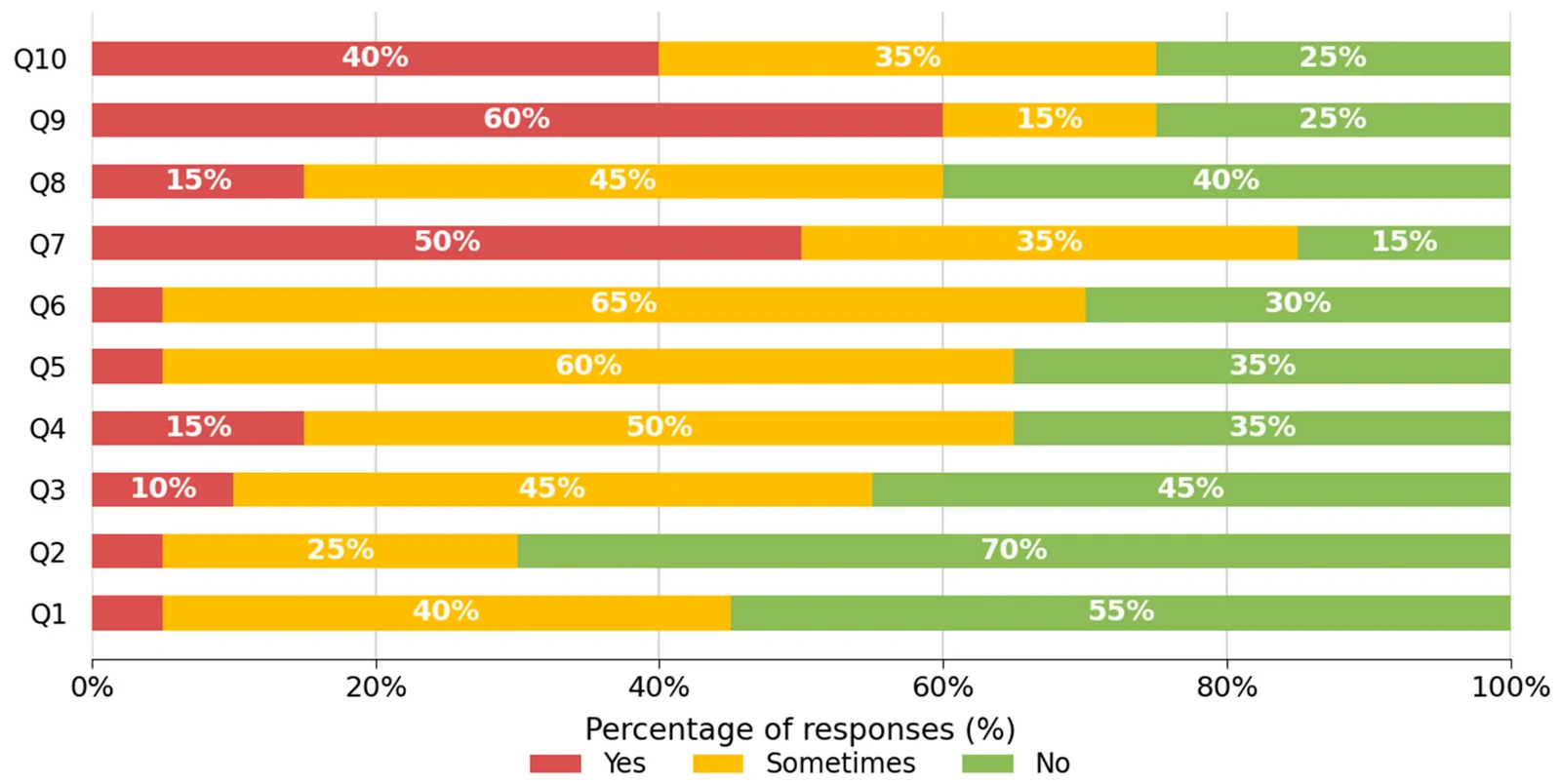
Distribution of FAI responses in the selected higher-score group (*n* = 20).

All ten FAI item scores were compared descriptively across the two selected groups (Table 6, Figure 2). Because the total FAI score used to select these participants contains all ten items, their between-group differences are expected to be interdependent.

The item comparisons describe the symptoms underlying selection into the two groups; Q7 and Q9 should not be interpreted as independent validation of a T-Scan association.

#### 3.2.4. Previous Orthodontic and Occlusal Dental Treatments

Among the 12 study-group participants with previous occlusal dental treatment, 2 reported that symptoms had been present before treatment, 5 reported symptom onset after treatment, and 5 could not specify the temporal relationship.

Previous dental treatment involving an occlusal surface was reported by 12/20 study participants and 5/20 controls (Table 7).

### 3.3. T-Scan III Findings in the Final Study and Control Groups

#### 3.3.1. Availability of T-Scan III Data

The analysed instrumental groups each contained 20 people. ID 17 had no usable T-Scan recording; protrusive movement data could not be calculated for ID 4, and the blocked lateral excursions of IDs 7 and 15 made the associated dynamic measures undefined. The left MI force entry for control ID 95 was excluded from the original side-specific comparison because its scale was initially unclear; its verified percentage value was used in the added bilateral deviation analysis. ID 83, who was outside the analysed control group, had three populated left-lateral T-Scan fields; the reason for their exclusion requires confirmation. Table 8 gives the variable-specific valid denominators. A missing recording and an uncomputable measure for an absent or blocked movement were distinguished; an undefined time was never coded as zero.

Available-case analyses used each measure’s valid denominator (Table 8), without assuming random missingness. In a sensitivity analysis, coding both blocked lateral movements as absence gave 9/19 versus 1/20 active premature contacts (*p* = 0.003; q = 0.020); coding both as presence gave 11/19 versus 1/20 (*p* < 0.001; q = 0.005). These analyses are separate from the primary 9/17 versus 1/20 comparison. Movement duration remained undefined in either coding.

#### 3.3.2. T-Scan III Findings in Maximum Intercuspation

Static right and left MI force fractions showed no clear group difference (Table 9). The original left-force analysis excluded the initially ambiguous-scale ID 95 entry and used 19 valid values per group; interpreting it as 52.45% after verification did not materially change that comparison (nominal *p* = 0.811 versus 0.770 on exclusion). The added individual absolute deviation from 50:50 was 8.74 ± 7.17 percentage points [*n* = 19] in the study group and 7.25 ± 5.30 [*n* = 20] in controls. The mean difference was 1.49 percentage points (Welch 95% CI −2.64 to 5.61; two-sided *p* = 0.469; q = 0.590; Table 9). MI occlusion time was 0.42 ± 0.14 s [*n* = 19] versus 0.28 ± 0.14 s [*n* = 20] (*p* < 0.001; q = 0.015).

#### 3.3.3. T-Scan III Findings During Protrusion

For protrusive movement, dynamic values were available for 18 study participants and 20 controls. Disclusion time was 0.66 ± 0.28 versus 0.42 ± 0.17 s (*p* = 0.003; q = 0.020). Other protrusive differences did not reach q < 0.05 in the 23-test group family (Table 9).

Incisal/anterior guidance predominated in both groups, whereas multiple contacts or incomplete disclusion were more often observed in the study group. Guidance type was analysed primarily descriptively because the low frequencies in some categories precluded robust inferential analysis.

#### 3.3.4. T-Scan III Findings During Right Lateral Excursion

During right lateral excursion, canine guidance was the most frequent guidance pattern in both groups, occurring in 14/19 valid study-group participants (73.68%) and 15/20 controls (75.00%). Anterolateral group guidance occurred in 2/19 (10.53%) and 3/20 (15.00%), respectively, and lateral group guidance in 1/19 (5.26%) and 2/20 (10.00%). Blocked guidance was observed only in the study group (2/19; 10.53%).

During right lateral excursion, active premature contacts occurred in 9/17 study participants and 1/20 controls (*p* = 0.002; q = 0.015; risk difference 47.9 percentage points, 95% CI 19.1–69.2). Other nominal differences should be read with their adjusted q values in Table 9.

**Figure 3:**
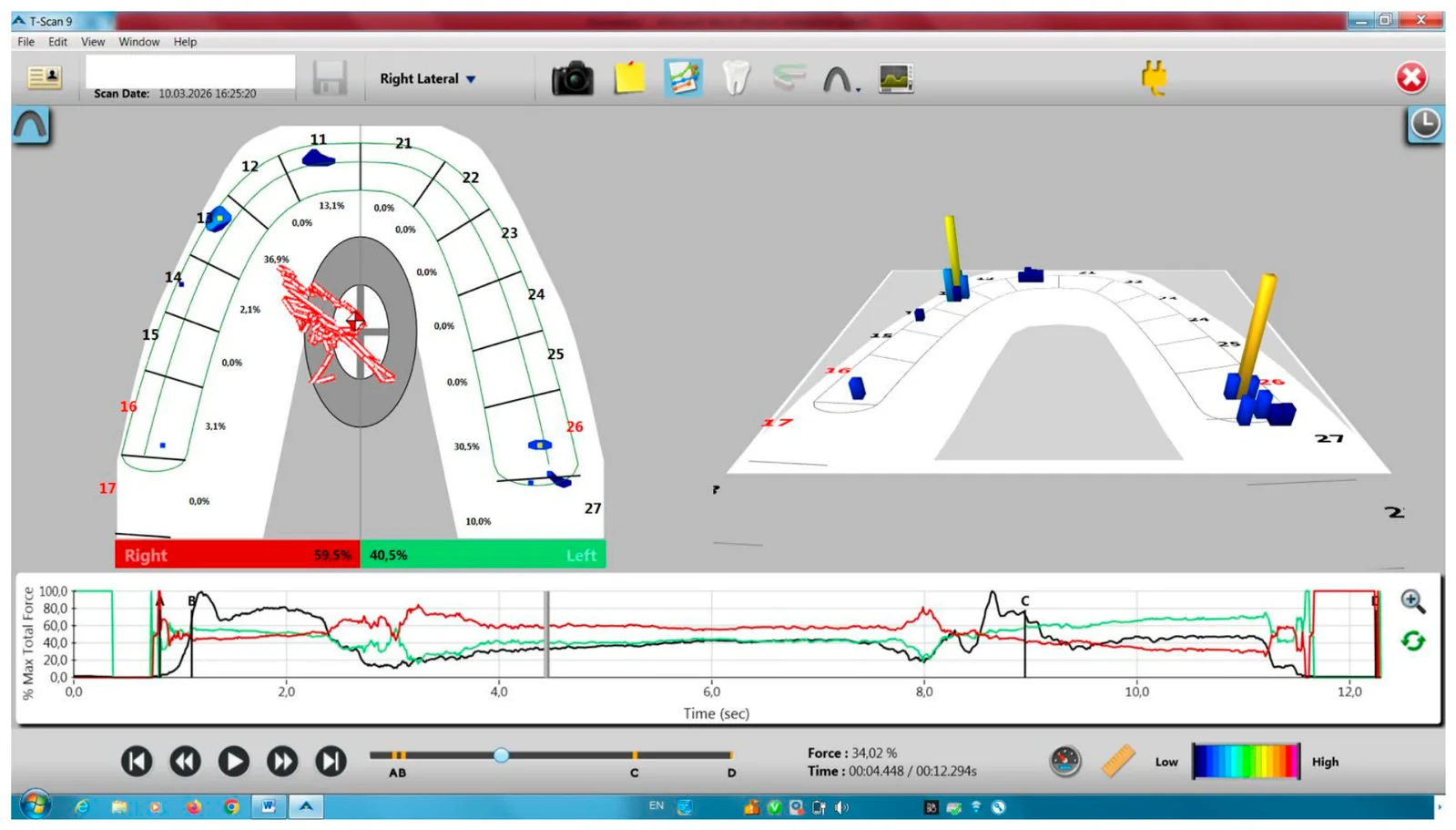
Right lateral movement, with active and inactive premature contacts.

**Figure 4:**
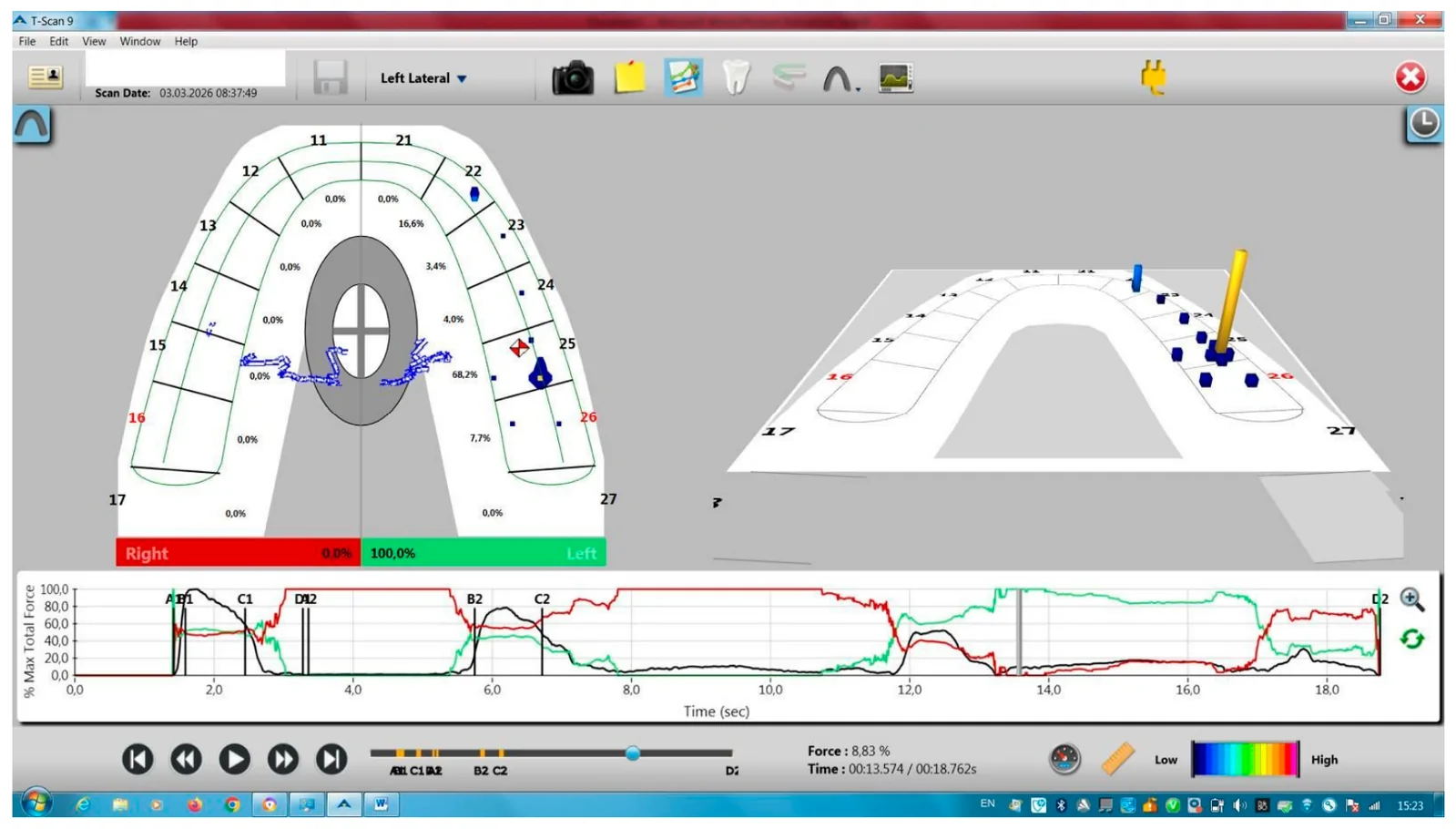
Left lateral movement with active premature contacts.

The guidance patterns are descriptive because several categories contain few observations. The lateral comparisons pertain only to the participants selected at opposite ends of the FAI distribution. Illustrative recordings are shown in Figure 3 and Figure 4.

#### 3.3.5. T-Scan III Findings During Left Lateral Excursion

During left lateral excursion, canine guidance was common in both analysed groups; the guidance-type entries are descriptive (Table 9). Two study participants had blocked movements, so dynamic left-lateral variables have 17 rather than 19 valid study values.

Active premature contacts during left lateral excursion occurred in 9/17 study participants and 1/20 controls (*p* = 0.002; q = 0.015; risk difference 47.9 percentage points, 95% CI 19.1–69.2). Active interferences occurred in 14/17 versus 9/20 (*p* = 0.040; q = 0.088), while inactive interferences occurred in 9/17 versus 9/20 (*p* = 0.746; q = 0.805). The previously reported 94.12% versus 47.37% and *p* = 0.006 are inconsistent with the individual data.

Left lateral disclusion time was 0.66 ± 0.18 s [*n* = 17] versus 0.52 ± 0.23 s [*n* = 20] (*p* = 0.019; q = 0.072). No undefined duration for a blocked movement was coded as zero. Figure 3 and Figure 4 provide examples of lateral excursion recordings.

### 3.4. Associations Between FAI Score and Occlusal Parameters

Spearman correlations between the corrected item-sum FAI score and occlusal measures were calculated using available pairs in the 40 selected participants (Table 10). Twenty-one correlations constituted a separate exploratory family; inference does not extend to the 103 screened students.

The strongest correlations involved active premature contacts during left lateral excursion (ρ = 0.578; *n* = 37; *p* < 0.001; q = 0.004), MI occlusion time (ρ = 0.534; *n* = 39; *p* < 0.001; q = 0.005), and active premature contacts during right lateral excursion (ρ = 0.524; *n* = 37; *p* < 0.001; q = 0.006).

These coefficients describe the selected upper-versus-lower FAI contrast. Selection on extreme scores can change both apparent effect magnitude and correlation; the unmeasured intermediate scores preclude estimation of the T-Scan relationship across the full screened cohort.

The correlation of protrusive inactive interference with the score was ρ = 0.328 (*n* = 38; *p* = 0.044; q = 0.082), a weak, nominal association that did not meet the declared false discovery rate threshold. The previously reported ρ = 0.326, *p* = 0.046 was classified inconsistently.

The complete set of 21 valid-pair counts, coefficients, bootstrap confidence intervals, nominal *p* values and adjusted q values appears in Table 10.

Several dynamic measures met q < 0.05, but neither their clinical significance nor their behaviour among intermediate FAI scorers can be inferred from this design.

### 3.5. Associations of Individual FAI Manifestations with T-Scan III Parameters

The Q7 and Q9 item analyses are exploratory: each item contributes to the total FAI score that determined selection into the two instrumental groups. These item associations are not independent of group membership.

#### 3.5.1. Association of TMJ Sounds (Q7) with T-Scan III Parameters

In the 40 selected participants, Q7 was positive in 17, all from the study group. Table 11 and Table 12 present seven continuous and 12 binary comparisons, respectively; together they form one 19-test Q7 family.

Q7 was associated, after adjustment, with longer MI occlusion time (nominal *p* = 0.003; q = 0.041) and active premature contacts during right and left lateral excursions (both *p* = 0.006; q = 0.041). Valid denominators and effect estimates are shown in Table 11 and Table 12.

No other Q7–T-Scan comparison remained below q = 0.05; Table 12 provides the underlying event counts and available denominators. These differences may reflect the selection procedure because no control reported Q7.

Q7 findings should not be taken as an independent symptom-specific mechanism: the Q7 response contributes to the FAI score used to assemble both groups.

#### 3.5.2. Association of Perceived Inadequate Occlusal Contact (Q9) with T-Scan III Parameters

Q9 was positive in 15 of the 40 selected participants, all in the study group. Table 13 and Table 14 report seven continuous and 12 binary comparisons using a separate 19-test Q9 family.

None of the Q9–T-Scan comparisons retained q < 0.05. The Q9 numerical results are distinct from Q7, as shown in Table 13 and Table 14; their earlier repetition arose from inconsistent reporting.

For example, left-lateral active premature contacts occurred in 6/13 participants with positive Q9 and 4/24 without it (two-sided Fisher *p* = 0.118; q = 0.404), rather than the previously reported *p* = 0.006. All counts in Table 14 were recalculated from the individual data.

The Q9 results are exploratory and dependent on FAI-based group selection; no independent Q9 association remained after multiplicity adjustment.

## 4. Discussion

TMD is recognised as a common cause of non-dental orofacial pain in the head and neck region. Together with back pain and headache, orofacial pain has been described among the most frequent chronic pain presentations [42].

The precise pathophysiology of TMD remains incompletely understood despite extensive experimental research, and several multifactorial models have been proposed [43,44,45,46].

Signs and symptoms associated with TMD often involve more than one component of the masticatory system [47]. The three major manifestations are pain, limited mandibular range of motion, and TMJ sounds. Pain is usually the predominant symptom, commonly involving the temporal and cheek regions as well as the periauricular area, and may be aggravated by chewing, speaking, and yawning. It may be intermittent or persistent and usually moderate, although severe pain can occur. Pain and tenderness on palpation of the pericranial muscles and TMJ are common and frequently coexist [48]. Other manifestations include comorbid pain conditions such as tension-type headache, neck and back pain, and psychosocial distress expressed as depression and anxiety [49].

In 2020, the International Headache Society published the first International Classification of Orofacial Pain. The manuscript cites this classification as defining TMD-related pain as pain localised to the TMJ occurring at rest, during jaw movement, or on palpation, without a known causal disorder [50].

TMD often requires input from several healthcare disciplines. Patients may consult multiple clinicians while seeking curative treatment, some interventions being costly, ineffective, or potentially iatrogenic (National Academies of Sciences, Engineering, and Medicine, 2020) [51]. Evidence also indicates substantial psychosocial impairment among patients with TMD, as identified using Axis II instruments within the Diagnostic Criteria for Temporomandibular Disorders [52]. These instruments have demonstrated reliability and validity for assessing distress, pain, and disability in patients with TMD [52].

The observed FAI screening proportion was 41.75% in this student sample after summing the item responses. It should not be equated with the prevalence of clinically confirmed TMD. The two instrumental groups were sampled at opposite ends of the FAI distribution, and several selected participants lacked computable dynamic recordings. These design features, the small effective denominators, and the absence of documented diagnostic confirmation restrict the generalisability and precision of the occlusal comparisons.

Several studies have assessed TMD prevalence using the FAI. Nomura et al. [53] reported a prevalence of 53.21% among 218 dental students in Ribeirão Preto, São Paulo, Brazil. Rehman et al. [54] reported 41.6% among 375 students at Liaquat National Hospital and Medical College in Karachi, Pakistan. A study of 1533 students at Hacettepe University in Ankara, Türkiye, reported 58.6% [55]. Budu et al. [56] reported an overall prevalence of 58.7% among 109 volunteers examined in Iași, Romania. Mitro et al. [33] reported that approximately 78% of dental students in Iași had at least one sign or symptom suggestive of TMD, supporting the usefulness of the FAI for initial assessment [33]. Shivamurthy et al. [57] identified a prevalence of 33.3% among 300 students at Ramaiah University of Applied Sciences in Bengaluru, India.

Shekhar et al. [58] studied patients with severe obstructive sleep apnoea in India and identified FAI symptoms suggestive of TMD in 52.7% of participants.

Silveira et al. [59] studied adults from the general population in Rio Grande do Sul, southern Brazil, and reported a TMD prevalence of 13.4%, with moderate and severe forms predominating [59].

To investigate the relationship between TMD and dental occlusion, the present study related FAI findings to T-Scan III occlusal analysis. T-Scan is widely used for digital static and dynamic occlusal assessment because it objectively records occlusal-force distribution, the sequence of dental contacts, and temporal occlusal parameters that cannot be obtained from conventional methods such as articulating paper [28,60]. It can record contact sequence and quantitative changes over time, identify areas of excessive occlusal force, and measure the interval from initial contact to completion of movement [28,61]. T-Scan has also been described as a useful tool for identifying occlusal interferences [62,63,64].

Research on the relationship between dental occlusion and TMD has evolved from simplified occlusion-centred models towards a multifactorial perspective in which occlusal factors represent only one of many components involved in TMD onset and persistence [15]. The association remains widely discussed [15,18], and current models have shifted from a strictly mechanistic interpretation towards a multidimensional, patient-phenotype-based framework [65].

Yaroslavska et al. [66] noted that some clinicians associate TMD with malocclusion, whereas others emphasise age-related features of the growing organism and morphological or psychomotor processes occurring around puberty [66].

Occlusion is therefore not currently regarded as a universal aetiological factor but rather as a potential biomechanical risk factor whose contribution may depend on individual susceptibility and interactions with other determinants of TMD [65].

In earlier decades, malocclusion and occlusal interferences were considered major causes of TMD, supporting widespread use of irreversible occlusal therapies [67]. This concept was based partly on observations that patients with TMD frequently exhibit occlusal discrepancies, reduced posterior support, interferences during mandibular movements, and differences between centric relation and MI.

Subsequent studies in the early 1990s identified occlusal characteristics proposed as TMD risk factors, including anterior open bite, unilateral posterior crossbite, overjet >6–7 mm, absence of five or more posterior teeth, and a centric relation–MI discrepancy >2 mm [11].

Authors cited by Pascu et al. [11] reported similar observations. Barrera-Mora [68] associated Angle class II and open bite with TMJ ligamentous hypermobility, while crossbite was described as a possible factor in TMD [68]. Perrotta, Pullinger, and Sonnesen likewise reported associations of open bite and unilateral or bilateral crossbite with joint dysfunction [11]. These findings contributed to the earlier concept of a direct relationship between malocclusion and TMD.

As research became more sophisticated, attention shifted from simple clinical associations towards biomechanical mechanisms. Okeson proposed that increasing discrepancy between the musculoskeletal stable condylar position and maximum intercuspation may increase the risk of intracapsular disorders. In this model, occlusal interferences require the condyle to leave a stable position to achieve dental occlusion, potentially generating muscle hyperactivity, altered intra-articular pressure, and structural changes over time [15]. Accordingly, occlusion should be considered in relation to the patient’s physiological adaptive capacity and as a potential risk factor rather than a single direct cause [13].

More recent data have also explored biomechanical plausibility. Zhu et al. [69] showed in silico that changing the location of occlusal contacts alters TMJ loading. Akkılıç and Balkaya [70] reported that chronic occlusal trauma may alter mandibular movement patterns, joint loading distribution, and TMJ function, suggesting biologically plausible mechanisms through which occlusion could contribute to temporomandibular symptoms.

Accumulating evidence nevertheless indicates that the relationship between occlusion and TMD is not linear. Okeson’s model introduced the concept of individual adaptive capacity [15]. Under this framework, an occlusal change, such as an inadequately adjusted new crown, may produce symptoms only when the resulting demand exceeds the patient’s capacity to adapt. Occlusal adjustment may then relieve symptoms without demonstrating that occlusion was the sole cause [15].

This perspective may explain why some studies report benefits after removal of occlusal interferences whereas others find weak or absent associations between occlusal factors and TMD. Longitudinal studies discussed by Okeson suggest that removal of interferences in relatively asymptomatic populations may reduce subsequent TMD symptoms [15]. Conversely, Lekaviciute and Kriauciunas [9] described contradictory evidence, with some studies reporting associations with malocclusion, bruxism, or tooth loss and others reporting weak or absent correlations. Chisnoiu [10] similarly argued that many occlusal parameters traditionally considered important make only a limited contribution to TMD development.

Occlusal stability remains a recurrent topic in the literature. Pascu et al. [11] concluded that increased overjet, deep overbite, and premature contacts may overload the TMJ and alter mandibular function. Manfredini et al. [71] reported the highest odds of TMJ pain for overbite ≥4 mm combined with clinically diagnosed bruxism and for overjet ≥5 mm; neither association was statistically significant, while Mélou (2024), as cited in [11], reported associations involving laterotrusive interferences and anterior overbite >4 mm. Gupta et al. [72] discussed findings linking TMD pain to combined anterior and posterior crossbite and posterior crossbite, while deep bite was linked to tooth wear; the review does not establish deep bite as a proven cause of TMD.

A systematic review by Manfredini et al. [73] contributed substantially to the current interpretation of the occlusion–TMD relationship. Across 25 clinical studies, most investigated occlusal characteristics showed no clinically relevant association with TMD. Of approximately 40 variables, only centric relation–maximum intercuspation discrepancy and mediotrusive interferences were relatively consistently associated with TMD [73].

A major conceptual shift has been recognition that occlusal changes may be consequences as well as potential causes of TMD. Caldas et al. [67] argued that many clinically observed occlusal changes are secondary to joint or muscle disorders and reflect adaptation of the stomatognathic system. This may explain why irreversible occlusal interventions do not consistently produce predictable outcomes and why joint and muscle assessment should precede definitive treatment [67]. Evidence-based literature generally considers orthodontic treatment neutral with respect to TMD, neither causing nor curing it [74,75]. Although occlusal characteristics have been proposed as a primary aetiology, the supporting evidence remains weak and inconclusive [76].

Building on these concepts, an international working group within the International Association for Dental, Oral, and Craniofacial Research developed consensus-based standards of care for TMD management [77]. Given the biopsychosocial and multifactorial aetiology of TMD, therapeutic decisions should be patient-centred and focus on symptom control, self-management, and reducing the impact on daily life [77]. Irreversible restorative treatment or alteration of occlusion or condylar position is not indicated for most TMD, except in specific circumstances involving acute or progressive occlusal changes related to condylar disorders [74,77].

Four dynamic T-Scan differences persisted after control of the false discovery rate in the declared group-comparison family: MI occlusion time, protrusive disclusion time, and active premature contacts during right and left lateral excursions. Some other differences were nominally significant but did not remain so after adjustment. Correlations with FAI score must be understood in the context of extreme-score selection, and the Q7/Q9 analyses overlap with the variables that determined that selection. The findings are therefore exploratory associations that warrant assessment in an independently sampled and clinically characterised cohort. Neither a causal contribution of occlusal measures to symptoms nor benefit from occlusal treatment follows from this cross-sectional comparison.

The static hemiarch force fractions did not distinguish the groups in the available-case comparisons. The recorded scale of the left-force value for ID 95 was initially unclear; interpreting it as the subsequently verified 52.45% in a separate sensitivity analysis did not materially change the nominal side-specific comparison. The additional individual deviation from 50:50 also showed no clear group difference (mean difference 1.49 percentage points, 95% CI −2.64 to 5.61).

Active premature lateral contacts were the most consistent between-group binary findings. The absolute differences and their confidence intervals convey the magnitude and uncertainty, without establishing clinical usefulness.

Dynamic durations were undefined for absent or blocked movements; analysing only recorded movements may affect the estimates. Coding both blocked lateral movements as absent or as present preserved the qualitative active-premature-contact contrast in a sensitivity analysis, but does not resolve why the movements were blocked.

Q7 and Q9 formed part of the selection score. The Q7 signals and the absence of Q9 signals after adjustment cannot be separated from the enriched contrast between screened groups.

T-Scan analysis can characterise the timing and distribution of occlusal contacts and identify interferences [78,79], whereas the Fonseca questionnaire assesses participant-reported symptoms [33]. In this regard, the two assessments provide complementary information without demonstrating a causal relationship. The present study examines the association of these parameters with symptomatology, without testing the efficacy of occlusal adjustment or the causal direction of the relationship.

The T-Scan sample comprised deliberately selected high- and low-scoring participants; intermediate scorers were not examined. Data absence, undefined movements, an initially inconsistent-scale left-force entry, small valid denominators, uncertain operational definitions and the absence of documented clinical diagnostic confirmation constrain interpretation. The reason for the exclusion of control-coded ID 83, despite three populated left-lateral fields, also needs clarification.

The design is cross-sectional and exploratory. These associations do not imply causation, establish diagnostic performance or support a therapeutic recommendation; their magnitude in the entire cohort cannot be inferred.

## 5. Conclusions

Frequently reported FAI manifestations included feeling tense or anxious, frequent headaches, and TMJ sounds during chewing or wide mouth opening, whereas difficulty with lateral mandibular movement and wide mouth opening were among the least frequent manifestations.

T-Scan III assessment showed that between-group differences were more apparent for dynamic parameters than for static occlusal-force distribution. The study group had longer occlusion/disclusion times and more frequent premature contacts, particularly during lateral movements.

The strongest association with Fonseca score was observed for active premature contacts during left lateral excursion, followed by active premature contacts during right lateral excursion and MI occlusion time.

The present exploratory findings support further study of occlusal parameters alongside TMD symptom screening, but do not establish a need for occlusal treatment in patients with nonspecific orofacial pain.

## Figures and Tables

**Table 1:** Demographic characteristics of the initial sample (*n* = 103).

Characteristic/Reported Measure	Total (*n* = 103)	Female Participants (*n* = 61)	Male Participants (*n* = 42)
Age, years, mean ± SD	24.15 ± 3.40	24.90 ± 4.04	23.05 ± 1.65
Age, years, range	21–45	21–45	21–29
Fonseca score, points, mean ± SD	17.14 ± 15.79	16.15 ± 16.79	18.57 ± 14.28
Fonseca score, points, median	15.00	10.00	15.00
Fonseca score, points, range	0–90	0–90	0–50

Note: SD = standard deviation; *n* = number of participants. Continuous variables are reported as mean ± SD, median, and minimum–maximum range.

**Table 2:** Distribution of FAI screening categories in the initial sample (*n* = 103).

FAI Screening Category (Item-Sum Score)	*n*	% of Sample	Female Participants *n* (% from Category)	Male Participants, *n* (%from Category)	FAI Score Range
No manifestations	60	58.25	38 (63.33%)	22 (36.67%)	0–15
Mild	36	34.95	18 (50.00%)	18 (50.00%)	20–40
Moderate	6	5.83	4 (66.67%)	2 (33.33%)	45–65
Severe	1	0.97	1 (100.00%)	0 (0.00%)	70–100
TOTAL	103	100.00	61 (59.22%)	42 (40.78%)	0–100

Note: percentages use 103 as the denominator, while sex percentages within rows use the category total. FAI categories are screening classifications. The fixed-margin Monte Carlo test (100,000 random tables) yielded *p* ≈ 0.530.

**Table 3:** Distribution of responses to Fonseca items Q1–Q10 in the initial sample (*n* = 103).

FAI Item	Yes		Sometimes		No		Positive Response	
	*n*	%	*n*	%	*n*	%	*n*	%
Q1-Difficulty opening the mouth widely	1	0.97	9	8.74	93	90.29	10	9.71
Q2-Difficulty moving the mandible from side to side	1	0.97	7	6.80	95	92.23	8	7.77
Q3-Pain/fatigue in the masticatory muscles during chewing	2	1.94	19	18.45	82	79.61	21	20.39
Q4-Frequent headaches	8	7.77	29	28.16	66	64.08	37	35.92
Q5-Neck pain or stiffness	3	2.91	32	31.07	68	66.02	35	33.98
Q6-Pain in the ear region or TMJ discomfort	2	1.94	20	19.42	81	78.64	22	21.36
Q7-TMJ sounds during chewing or wide mouth opening	19	18.45	18	17.48	66	*64.08*	37	35.92
Q8-Habit of clenching or grinding the teeth	8	7.77	27	26.21	68	66.02	35	33.98
Q9-Perception that the teeth do not contact correctly	14	13.59	7	6.80	82	79.61	21	20.39
Q10-Feeling tense or anxious	15	14.56	39	37.86	49	47.57	54	52.43

Note: positive response = Yes + Sometimes; *n* = number of participants; % = percentage of the initial sample. Scoring: No = 0, Sometimes = 5, Yes = 10.

**Table 4:** Comparative characteristics of the final groups (20 vs. 20).

Characteristic/Reported Measure	Study Group (*n* = 20)	Control Group (*n* = 20)	Final Groups, Total (*n* = 40)	*p*-Value
Sex, *n* (%)	7 female (35.00%)/13 male (65.00%)	14 female (70.00%)/6 male (30.00%)	21 female/19 male	*p* = 0.056 *
Age, years, mean ± SD	23.50 ± 2.21	24.65 ± 4.12	24.08 ± 3.32	*p* = 0.368 **
Age, years, range	21–28	21–38	21–38	--
Fonseca score, mean ± SD	41.75 ± 13.40	0.75 ± 1.83	21.25 ± 22.81	*p* < 0.001 **
Fonseca score, median	37.50	0.00	15.00	--

Note: percentages use 20 per group; age and FAI comparisons use two-sided Mann–Whitney U tests, and sex uses two-sided Fisher exact testing. FAI totals for selected IDs 10 and 19 were reconciled with their confirmed item responses. *—the two-sided Fisher’s exact test for the comparison of sex distribution. **—the two-sided Mann–Whitney U test for the comparisons of age and FAI score. FAI = Fonseca Anamnestic Index. SD = standard deviation. *n* = number of participants. FAI totals for selected participants with IDs 10 and 19 were reconciled with their confirmed item responses.

**Table 5:** Distribution of FAI screening categories in the final groups.

FAI Screening Category	Study Group, *n* (%)	Control Group, *n* (%)	Total, *n* (%)	Fonseca Score, Mean ± SD
No FAI-defined manifestations	0 (0.00%)	20 (100.00%)	20 (50.00%)	0.75 ± 1.83
Mild FAI manifestations	13 (65.00%)	0 (0.00%)	13 (32.50%)	35.00 ± 4.08
Moderate FAI manifestations	6 (30.00%)	0 (0.00%)	6 (15.00%)	48.33 ± 2.58
Severe FAI manifestations	1 (5.00%)	0 (0.00%)	1 (2.50%)	90.00

Note: *n* = number of participants; % = within-group percentage; SD = standard deviation.

**Table 6:** Comparison of Q1–Q10 item scores between the study and control groups.

FAI Item	Study Group, Mean ± SD	Control Group, Mean ± SD	*p*
Q1-Difficulty opening the mouth widely	2.50 ± 3.03	0.00 ± 0.00	<0.001
Q2-Difficulty moving the mandible from side to side	1.75 ± 2.94	0.00 ± 0.00	0.009
Q3-Pain/fatigue in the masticatory muscles whilst chewing	3.25 ± 3.35	0.00 ± 0.00	<0.001
Q4-Frequent headaches	4.00 ± 3.48	0.00 ± 0.00	<0.001
Q5-Neck pain or stiffness	3.50 ± 2.86	0.00 ± 0.00	<0.001
Q6-Pain in the ear region or TMJ discomfort	3.75 ± 2.75	0.00 ± 0.00	<0.001
Q7-TMJ sounds during chewing or wide mouth opening	6.75 ± 3.73	0.00 ± 0.00	<0.001
Q8-The habit of clenching or grinding one’s teeth	3.75 ± 3.58	0.00 ± 0.00	<0.001
Q9-Perception that the teeth do not contact correctly	6.75 ± 4.38	0.00 ± 0.00	<0.001
Q10-Feeling tense or anxious	5.75 ± 4.06	0.75 ± 1.83	<0.001

Note: SD = standard deviation; *p* = two-sided *p*-value. Item score: 0 = No, 5 = Sometimes, 10 = Yes. Mann–Whitney U test.

**Table 7:** Distribution of participants with previous occlusal alterations by group.

Variable	Study	Control	Risk Difference (95% CI)	*p*	q
Previous orthodontic treatment	9/20 (45.0%)	5/20 (25.0%)	RD 20.0 (−9.1 to 45.0) pp	0.320	0.446
Previous treatment of occlusal surfaces	12/20 (60.0%)	5/20 (25.0%)	RD 35.0 (4.4 to 57.8) pp	0.054	0.088

Note: risk difference is study minus control proportion, with a 95% interval; *p* values are two-sided Fisher exact tests; q values use the 23-test exploratory group-comparison family.

**Table 8:** Number of valid cases for T-Scan parameters.

T-Scan Component	Study Group, Valid Values *n*/*N*	Control Group, Valid Values *n*/*N*	Explanation of Missing Values
MI—right force and times	19/20	20/20	ID 17: no usable recording
MI—left force	19/20	19/20	ID 17: no usable recording; the initially ambiguous-scale control value was excluded from the original left-force comparison
MI—absolute deviation from 50:50	19/20	20/20	Valid bilateral percentage values after scale verification
Protrusive guidance	19/20	20/20	ID 17: no usable recording
Protrusive contacts/interferences/disclusion time	18/20	20/20	ID 4: no protrusive movement; ID 17: no usable recording
Right lateral guidance type	19/20	20/20	ID 17: no usable recording
Right lateral contacts/interferences/disclusion time	17/20	20/20	IDs 7 and 15: blocked movement; ID 17: no usable recording
Left lateral guidance type	19/20	20/20	ID 17: no usable recording
Left lateral contacts/interferences/disclusion time	17/20	20/20	IDs 7 and 15: blocked/undefined; ID 17: no usable recording

Note: denominators are out of 20 per analysed group. ID 17 has no usable recording; IDs 4, 7 and 15 have movement-specific undefined values. The original left-force comparison excluded the initially ambiguous-scale value for control ID 95, yielding 19/20 controls; after verification, the added bilateral deviation analysis included 20/20 controls. ID 83 is outside the analysed controls despite three populated left-lateral fields.

**Table 9:** Comparison of T-Scan parameters between the study and control groups.

Parameter	Study	Control	Effect (95% CI)	*p*	q
MI right force fraction	0.48 ± 0.11 [19]	0.50 ± 0.09 [20]	r_rβ_ −0.05 (−0.41 to 0.32)	0.811	0.811
MI left force fraction	0.52 ± 0.11 [19]	0.50 ± 0.09 [19]	r_rβ_ 0.06 (−0.34 to 0.45)	0.770	0.805
MI absolute deviation from 50:50 (percentage points)	8.74 ± 7.17 [19]	7.25 ± 5.30 [20]	Mean difference 1.49 (−2.64 to 5.61) pp	0.469	0.590
MI occlusion time (s)	0.42 ± 0.14 [19]	0.28 ± 0.14 [20]	r_rβ_ 0.62 (0.34 to 0.88)	<0.001	0.015
MI disclusion time (s)	0.44 ± 0.16 [19]	0.38 ± 0.30 [20]	r_rβ_ 0.41 (0.05 to 0.73)	0.028	0.072
Protrusive guidance present	18/19 (94.7%)	20/20 (100.0%)	RD −5.3 (−24.6 to 11.4) pp	0.487	0.590
Protrusion: active interference	14/18 (77.8%)	12/20 (60.0%)	RD 17.8 (−11.5 to 42.9) pp	0.307	0.446
Protrusion: inactive interference	14/18 (77.8%)	9/20 (45.0%)	RD 32.8 (1.8 to 56.1) pp	0.052	0.088
Protrusion: active premature contact	11/18 (61.1%)	5/20 (25.0%)	RD 36.1 (4.7 to 59.3) pp	0.047	0.088
Protrusion: inactive premature contact	8/18 (44.4%)	2/20 (10.0%)	RD 34.4 (6.2 to 57.4) pp	0.027	0.072
Protrusive disclusion time (s)	0.66 ± 0.28 [18]	0.42 ± 0.17 [20]	r_rβ_ 0.56 (0.24 to 0.82)	0.003	0.020
Right excursion: active interference	16/17 (94.1%)	12/20 (60.0%)	RD 34.1 (6.3 to 56.0) pp	0.023	0.072
Right excursion: inactive interference	10/17 (58.8%)	8/20 (40.0%)	RD 18.8 (−12.4 to 45.5) pp	0.330	0.446
Right excursion: active premature contact	9/17 (52.9%)	1/20 (5.0%)	RD 47.9 (19.1 to 69.2) pp	0.002	0.015
Right excursion: inactive premature contact	4/17 (23.5%)	3/20 (15.0%)	RD 8.5 (−16.7 to 34.2) pp	0.680	0.782
Right disclusion time (s)	0.69 ± 0.23 [17]	0.53 ± 0.26 [20]	r_rβ_ 0.39 (0.04 to 0.71)	0.043	0.088
Left excursion: active interference	14/17 (82.4%)	9/20 (45.0%)	RD 37.4 (6.1 to 59.7) pp	0.040	0.088
Left excursion: inactive interference	9/17 (52.9%)	9/20 (45.0%)	RD 7.9 (−22.3 to 36.3) pp	0.746	0.805
Left excursion: active premature contact	9/17 (52.9%)	1/20 (5.0%)	RD 47.9 (19.1 to 69.2) pp	0.002	0.015
Left excursion: inactive premature contact	7/17 (41.2%)	1/20 (5.0%)	RD 36.2 (9.2 to 59.4) pp	0.014	0.064
Left disclusion time (s)	0.66 ± 0.18 [17]	0.52 ± 0.23 [20]	r_rβ_ 0.46 (0.10 to 0.77)	0.019	0.072
Right lateral excursion-guidance type: absent/blocked	2/19 (10.53%)	0/20 (0.00%)	—	—	—
Right lateral excursion-guidance type: canine	14/19 (73.68%)	15/20 (75.00%)	—	—	—
Right lateral excursion-guidance type: anterolateral group	2/19 (10.53%)	3/20 (15.00%)	—	—	—
Right lateral excursion-guidance type: lateral group	1/19 (5.26%)	2/20 (10.00%)	—	—	—
Left lateral excursion-guidance type: absent/blocked	2/19 (10.53%)	0/20 (0.00%)	—	—	—
Left lateral excursion-guidance type: canine	16/19 (84.21%)	17/20 (85.00%)	—	—	—
Left lateral excursion-guidance type: anterolateral group	0/19 (0.00%)	2/20 (10.00%)	—	—	—
Left lateral excursion-guidance type: lateral group	1/19 (5.26%)	1/20 (5.00%)	—	—	—

Note: brackets give the valid *n* for continuous variables; binary cells give event/valid *N*. Effect estimates are rank-biserial correlation or risk difference, with 95% confidence intervals; the added force-deviation comparison gives a mean difference (percentage points) with a Welch 95% confidence interval. *p-values are two-sided Mann–Whitney U or Fisher exact, except for the added two-sided Welch test; q-values* use the 23-test Benjamini–Hochberg family. Guidance types are descriptive. The original side-specific left-force comparison excluded the initially ambiguous scale value for ID 95; treating it as the subsequently verified 52.45% gave a sensitivity *p*-value of 0.811. The added deviation comparison used the confirmed bilateral percentage values. Absent/blocked movement durations are undefined. For lateral excursions, “active” denotes contacts and interferences on the working side (the side towards which the mandible moves), and “inactive” denotes those on the non-working side. These labels describe contact location and do not refer to neuromuscular activity.

**Table 10:** Spearman correlations between Fonseca score and T-Scan parameters/auxiliary variables.

Parameter	*n*	Spearman ρ	Bootstrap 95% CI	*p*	q
Left excursion: active premature contact	37	0.578	0.32 to 0.79	<0.001	0.004
Right excursion: active premature contact	37	0.524	0.23 to 0.75	<0.001	0.006
MI occlusion time (s)	39	0.534	0.27 to 0.76	<0.001	0.005
Left excursion: inactive premature contact	37	0.477	0.16 to 0.72	0.003	0.015
Protrusive disclusion time (s)	38	0.440	0.18 to 0.66	0.006	0.017
Left excursion: active interference	37	0.463	0.21 to 0.68	0.004	0.015
Right excursion: active interference	37	0.458	0.23 to 0.65	0.004	0.015
Left disclusion time (s)	37	0.377	0.05 to 0.65	0.022	0.0502
Protrusion: inactive premature contact	38	0.335	0.03 to 0.61	0.039	0.082
Protrusion: active premature contact	38	0.325	0.03 to 0.62	0.047	0.082
Previous treatment of occlusal surfaces	40	0.378	0.07 to 0.66	0.016	0.042
Right disclusion time (s)	37	0.295	0.00 to 0.55	0.076	0.114
Protrusion: inactive interference	38	0.328	0.04 to 0.59	0.044	0.082
MI disclusion time (s)	39	0.298	−0.03 to 0.61	0.066	0.106
Right excursion: inactive interference	37	0.233	−0.09 to 0.53	0.166	0.232
Protrusion: active interference	38	0.179	−0.12 to 0.48	0.282	0.369
Right excursion: inactive premature contact	37	0.096	−0.24 to 0.43	0.574	0.669
Left excursion: inactive interference	37	0.059	−0.26 to 0.38	0.729	0.766
MI right force fraction	39	−0.047	−0.36 to 0.28	0.776	0.776
MI left force fraction	38	0.058	−0.28 to 0.36	0.728	0.766
Previous orthodontic treatment	40	0.100	−0.20 to 0.43	0.540	0.667

Note: each *n* is the number of valid score–measure pairs. Spearman ρ has a percentile bootstrap 95% CI; *p* values are two-sided, and q values use the 21-test Benjamini–Hochberg family. Coefficients from extreme-score sampling are not population correlations. For lateral excursions, “active” denotes contacts and interferences on the working side (the side towards which the mandible moves), and “inactive” denotes those on the non-working side. These labels describe contact location and do not refer to neuromuscular activity.

**Table 11:** Comparison of T-Scan parameters between participants with positive and absent Q7 responses in the final sample (*n* = 40). Mann–Whitney U test.

Parameter	Positive Median [*n*]	Absent Median [*n*]	Rank-Biserial r (95% CI)	*p*	q
MI occlusion time (s)	0.400 [17]	0.240 [22]	r_rβ_ 0.55 (0.24 to 0.83)	0.003	0.041
Left disclusion time (s)	0.680 [15]	0.460 [22]	r_rβ_ 0.49 (0.14 to 0.77)	0.013	0.062
Protrusive disclusion time (s)	0.585 [16]	0.430 [22]	r_rβ_ 0.39 (0.04 to 0.70)	0.041	0.112
Right disclusion time (s)	0.600 [15]	0.540 [22]	r_rβ_ 0.22 (−0.16 to 0.58)	0.259	0.378
MI right force fraction	0.486 [17]	0.498 [22]	r_rβ_ 0.01 (−0.36 to 0.41)	0.977	1.000
MI left force fraction	0.514 [17]	0.494 [21]	r_rβ_ 0.00 (−0.37 to 0.38)	1.000	1.000
Force asymmetry (%)	12.600 [17]	12.800 [21]	r_rβ_ 0.12 (−0.25 to 0.47)	0.547	0.743

Note: Q7 positive means Sometimes/Yes; absence means No. Cells display median and valid *n*. Rank-biserial effects include bootstrap 95% CIs; two-sided Mann–Whitney *p* and 19-test family q values are reported.

**Table 12:** Association between Q7 and binary T-Scan variables in the final sample (*n* = 40). Fisher’s exact test.

Parameter	Positive	Absent	Risk Difference (95% CI)	*p*	q
Left excursion: active premature contact	8/15 (53.3%)	2/22 (9.1%)	RD 44.2 (14.4 to 67.1) pp	0.006	0.041
Right excursion: active premature contact	8/15 (53.3%)	2/22 (9.1%)	RD 44.2 (14.4 to 67.1) pp	0.006	0.041
Left excursion: active interference	13/15 (86.7%)	10/22 (45.5%)	RD 41.2 (9.6 to 62.1) pp	0.016	0.062
Protrusion: active premature contact	10/16 (62.5%)	6/22 (27.3%)	RD 35.2 (3.5 to 58.9) pp	0.047	0.112
Left excursion: inactive premature contact	6/15 (40.0%)	2/22 (9.1%)	RD 30.9 (3.4 to 56.0) pp	0.042	0.112
Right excursion: active interference	14/15 (93.3%)	14/22 (63.6%)	RD 29.7 (1.2 to 51.1) pp	0.056	0.118
Protrusion: inactive premature contact	7/16 (43.8%)	3/22 (13.6%)	RD 30.1 (1.6 to 54.8) pp	0.062	0.118
Protrusion: active interference	13/16 (81.2%)	13/22 (59.1%)	RD 22.2 (−7.8 to 45.9) pp	0.178	0.288
Left excursion: inactive interference	8/15 (53.3%)	10/22 (45.5%)	RD 7.9 (−22.7 to 36.5) pp	0.743	0.831
Right excursion: inactive interference	8/15 (53.3%)	10/22 (45.5%)	RD 7.9 (−22.7 to 36.5) pp	0.743	0.831
Protrusion: inactive interference	12/16 (75.0%)	11/22 (50.0%)	RD 25.0 (−6.2 to 49.3) pp	0.182	0.288
Right excursion: inactive premature contact	2/15 (13.3%)	5/22 (22.7%)	RD −9.4 (−32.2 to 18.2) pp	0.677	0.831

Note: events/valid *N* are reported separately by response group; risk differences have 95% CIs. Fisher exact *p* values are two-sided, with Benjamini–Hochberg q values across seven continuous and 12 binary Q7 comparisons. For lateral excursions, “active” denotes contacts and interferences on the working side (the side towards which the mandible moves), and “inactive” denotes those on the non-working side. These labels describe contact location and do not refer to neuromuscular activity.

**Table 13:** Comparison of T-Scan parameters between participants with positive and absent Q9 responses in the final sample (*n* = 40). Mann–Whitney U test.

Parameter	Positive Median [*n*]	Absent Median [*n*]	Rank-Biserial r (95% CI)	*p*	q
MI occlusion time (s)	0.400 [14]	0.240 [24]	r_rβ_ 0.49 (0.13 to 0.76)	0.013	0.158
Left disclusion time (s)	0.660 [13]	0.485 [26]	r_rβ_ 0.27 (−0.11 to 0.63)	0.186	0.477
Protrusive disclusion time (s)	0.520 [13]	0.430 [24]	r_rβ_ 0.31 (−0.07 to 0.64)	0.127	0.404
Right disclusion time (s)	0.600 [13]	0.595 [26]	r_rβ_ 0.21 (−0.16 to 0.56)	0.301	0.477
MI right force fraction	0.464 [14]	0.506 [24]	r_rβ_ −0.19 (−0.54 to 0.19)	0.327	0.477
MI left force fraction	0.536 [14]	0.492 [26]	r_rβ_ 0.20 (−0.20 to 0.57)	0.311	0.477
Force asymmetry (%)	16.300 [14]	12.100 [26]	r_rβ_ 0.08 (−0.28 to 0.46)	0.683	0.749

Note: Q9 positive means Sometimes/Yes; absence means No. Cells display median and valid *n*. Rank-biserial effects include bootstrap 95% CIs; two-sided Mann–Whitney *p* and 19-test family q values are reported.

**Table 14:** Association between Q9 and binary T-Scan variables in the final sample (*n* = 40). Fisher’s exact test.

Parameter	Positive	Absent	Risk Difference (95% CI)	*p*	q
Left excursion: active premature contact	6/13 (46.2%)	4/24 (16.7%)	RD 29.5 (−0.4 to 56.1) pp	0.118	0.404
Right excursion: active premature contact	7/13 (53.8%)	3/24 (12.5%)	RD 41.3 (10.5 to 65.7) pp	0.017	0.158
Left excursion: active interference	10/13 (76.9%)	13/24 (54.2%)	RD 22.8 (−9.8 to 47.0) pp	0.288	0.477
Protrusion: active premature contact	8/13 (61.5%)	8/25 (32.0%)	RD 29.5 (−3.0 to 55.0) pp	0.098	0.404
Left excursion: inactive premature contact	4/13 (30.8%)	4/24 (16.7%)	RD 14.1 (−12.3 to 42.8) pp	0.413	0.561
Right excursion: active interference	12/13 (92.3%)	16/24 (66.7%)	RD 25.6 (−4.2 to 46.6) pp	0.119	0.404
Protrusion: inactive premature contact	4/13 (30.8%)	6/25 (24.0%)	RD 6.8 (−19.8 to 36.4) pp	0.709	0.749
Protrusion: active interference	10/13 (76.9%)	16/25 (64.0%)	RD 12.9 (−18.5 to 37.4) pp	0.486	0.596
Left excursion: inactive interference	6/13 (46.2%)	12/24 (50.0%)	RD −3.8 (−33.4 to 27.1) pp	1.000	1.000
Right excursion: inactive interference	8/13 (61.5%)	10/24 (41.7%)	RD 19.9 (−12.6 to 46.8) pp	0.313	0.477
Protrusion: inactive interference	9/13 (69.2%)	14/25 (56.0%)	RD 13.2 (−18.7 to 39.4) pp	0.501	0.596
Right excursion: inactive premature contact	4/13 (30.8%)	3/24 (12.5%)	RD 18.3 (−7.6 to 46.3) pp	0.213	0.477

Note: events/valid *N* are reported separately by Q9 response group; risk differences have 95% CIs. Two-sided Fisher exact *p* and Benjamini–Hochberg q values are given across the 19 Q9 comparisons. For lateral excursions, “active” denotes contacts and interferences on the working side (the side towards which the mandible moves), and “inactive” denotes those on the non-working side. These labels describe contact location and do not refer to neuromuscular activity.

## Data Availability

The authors declare that the data of this research are available from the corresponding authors upon reasonable request.

## Abbreviations

The following abbreviations are used in this manuscript:

TMD	Temporomandibular dysfunction
FAI	Fonseca Anamnestic Index
TMJ	temporomandibular joint
MI	maximum intercuspation
